# Social Capital and Dietary Intakes Following the 2011 Great East Japan Earthquake and Tsunami

**DOI:** 10.2188/jea.JE20170117

**Published:** 2019-03-05

**Authors:** Sayuri Goryoda, Nobuo Nishi, Haruki Shimoda, Yuki Yonekura, Kiyomi Sakata, Seiichiro Kobayashi, Akira Ogawa, Ichiro Kawachi

**Affiliations:** 1 The Disease Prevention Science Course, Graduate School of Medical and Dental Science, Tokyo Medical and Dental University, Tokyo, Japan; 2 International Center for Nutrition and Information, National Institute of Health and Nutrition, National Institutes of Biomedical Innovation, Health and Nutrition, Tokyo, Japan; 3 Department of Hygiene and Preventive Medicine, Iwate Medical University School of Medicine, Iwate, Japan; 4 Graduate School of Nursing Science, St. Luke’s International University, Tokyo, Japan; 5 Iwate Medical University, Iwate, Japan; 6 Department of Social and Behavioral Sciences, Harvard T.H. Chan School of Public Health, Boston, MA, USA

**Keywords:** earthquake, social capital, social factor, dietary intake, Japan

## Abstract

**Background:**

Previous studies have identified poor dietary intake as a health risk affecting survivors of the 2011 Great East Japan Earthquake and Tsunami. We examined the association between different social factors (eg, living conditions and perceptions of community social capital) and dietary intakes among disaster-affected survivors.

**Methods:**

We studied 6,724 survivors in four municipalities of Iwate Prefecture 3 years after the disaster. Social capital was assessed via four items inquiring about respondents’ perceptions of social cohesion in their communities. Good dietary intake was defined according to the following criteria: intake of staple food ≥three times a day; intake of meat, fish and shellfish eggs, or soybean products ≥twice a day; vegetable intake ≥twice a day; and intake of fruit or dairy products ≥once a day. An individual who did not meet any of these criteria was defined as having poor dietary intake. We adjusted for covariates, including socioeconomic status, marital status, and residential area.

**Results:**

Poor dietary intake was reported by 31.6% of respondents. Poisson regression analyses revealed that the following factors were related to poor dietary intake: age <65 years (men: prevalence ratio [PR] 1.48; 95% confidence interval [CI], 1.29–1.71 and women: PR 1.55; 95% CI, 1.36–1.77), difficulties in living conditions (men: PR 1.18; 95% CI, 1.00–1.39 and women: PR 1.19; 95% CI, 1.01–1.40), and low perceptions of community social capital (women: PR 1.20; 95% CI, 1.04–1.38).

**Conclusions:**

Our findings suggest that social capital plays a role in promoting healthy dietary intake among women in disaster-affected areas.

## INTRODUCTION

Food shortages and disruptions to the food supply can affect dietary intakes in the aftermath of a natural disaster.^1^^,^^2^ The Great East Japan Earthquake and Tsunami of March 11, 2011 caused widespread destruction of retail services and transport infrastructure, which adversely affected the food supply in the affected region.^3^ Previous studies have focused on relationships between dietary intakes and various social factors, such as living conditions and housing arrangements of survivors.^4^^–^^6^ Living in evacuation shelters or temporary housing was associated with poor dietary intake.^4^^,^^5^ A previous study reported that in the year following the 2011 earthquake, women and older survivors were more likely to report a healthy dietary pattern, characterized by high intakes of fish and shellfish, soybean products, vegetables, fruit, and dairy products. High consumption of meat and eggs and was more prevalent in younger-aged survivors and men.^4^ In another survey conducted in 2012, evacuees living in temporary housing were found to have low dietary intakes of fruits and vegetables, meat, soybean products, and dairy products.^6^

Several factors could contribute to deteriorating nutritional habits in the aftermath of disaster, including worse access to food outlets due to residential displacement, financial difficulties, and social isolation. Social capital (SC), defined as “the resources that are accessed by individuals as a result of their membership of network or a group”, is believed to be particularly relevant for disaster resilience.^7^ When a major disaster strikes a community, recovery often depends on the mutual support of residents. The exchange of resources (eg, water, food, and clothing, as well as emotional support) constitutes a form of “informal insurance”, when more formal mechanisms of insurance fail.^8^

However, there have been no reports on the association between SC and the dietary intakes of residents affected by the 2011 Great East Japan Earthquake. On the basis of the hypothesis that the stock of community SC is an important determinant of disaster resilience, we sought to examine the associations between poor dietary intake and social factors, including perceptions of community SC, among survivors of the 2011 earthquake.

## METHODS

### Study population

**Figure 1.**
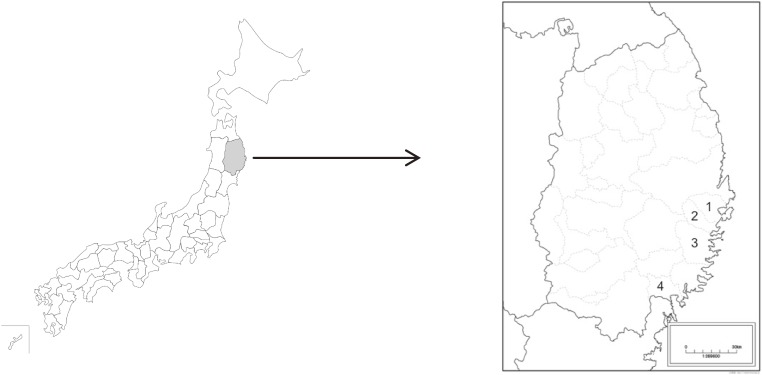
Location of Iwate prefecture, Japan. Damaged areas: 1.Yamada, 2.Otsuchi, 3.Kamaishi, and 4.Rikuzentakata.

The study population included 7,136 people aged 18 years or older who participated in a health survey for Great East Japan Earthquake survivors 3 years after the disaster. They were selected in severely affected regions of (Figure 1), where there was a considerable amount of property damage and lives lost. The survey was carried out from September 2011, when most of the survivors had moved from emergency shelters into temporary housing, and surveys were mailed to survivors in four municipalities.

Table 1 summarizes the characteristics of the study regions. In the first year of the survey, 3,216 people in the town of Yamada, 2,079 in the town of Otsuchi, 272 in the city of Kamaishi, and 4,908 in the city of Rikuzentakata participated in the survey. The number of people in each of these four areas whose death were directly or indirectly caused by the earthquake by the end of 2015 was 687, 854, 992, and 1,602, respectively. The number of partially or completely destroyed houses in our four sampled communities numbered 3,167, 4,167, 3,656, and 4,044, respectively. The present study was based on the third follow-up survey, and the number of participants from each of the four areas was 2,223 (69.1%), 1,492 (71.8%), 160 (58.8%), and 3,261 (66.4%), respectively.

**Table 1.** Characteristics of study regions

	Yamada	Otsuchi	Kamaishi	Rikuzentakata
Population before earthquake^a^	18,506	15,222	39,399	23,221
Number of people who died^b^	604	803	888	1,555
Number of houses destroyed^c^	3,167	3,717	3,648	3,341

^a^Population before earthquake of all ages. Office of International Affairs, Department of Policy and Regional Affairs, Iwate Prefecture.

^b^Number of people of all ages whose deaths were directly and indirectly caused by the earthquake by the end of 2012. Comprehensive Disaster Prevention Room, General Affairs Department, Iwate Prefecture.

^c^Number of houses destroyed included completely and partially destroyed homes.

### Dietary intakes

Regarding dietary intakes, we inquired about the frequency of food consumption during the previous several days, based on the Japanese food guidelines established by the Ministry of Health, Labour and Welfare.^9^ We asked about the frequency of consumption of the following food groups^4^^,^^10^: staple foods (rice, bread, and noodles), meat, fish and shellfish, eggs, soybean products (tofu and natto [fermented soybeans]), vegetables, fruit, and dairy products (milk, yogurt, and cheese). The response categories were none (<once a day), once a day, twice a day, three times a day, and four times or more a day. We categorized participants who fulfilled the following criteria as having good dietary intake: staple food items ≥three times a day; meat, fish and shellfish, eggs, or soybean products ≥twice a day; vegetables ≥twice a day; and fruit or dairy products ≥once a day. All remaining participants who did not meet these criteria were categorized as having poor dietary intake. Our index of “poor dietary intake” has been previously shown to be associated with mortality risk in a cohort study setting.^11^

### Social capital

We also inquired about residents’ perceptions of SC in the community. Our SC measure comprised four questions: (i) “most people in this neighborhood are likely to help each other”; (ii) “most people who live in this neighborhood can be trusted”; (iii) “most people in this neighborhood are likely to greet one another”; and (iv) “if there was any problem in this community, how likely is it that people will cooperate to try to solve the problem?”. Each question was scored from 1 to 5 (1 = “very high,” 2 = “high,” 3 = “average,” 4 = “low,” and 5 = “very low”). The total score ranged from 4 to 20. We used a cut-off point of ≤10 as the group with low SC.

### Living conditions

Socioeconomic circumstances were assessed by asking the question: “How do you feel about your current economic situation?”. Participants were requested to select an answer from “severe”, “difficult”, “rather difficult”, and “acceptable”.^4^

### Housing

We inquired about housing as follows: “At present, what kind of house do you live in?”. Each respondent chose one of five response options: (i) “the same as before the earthquake”, (ii) “living in temporary housing provided by the municipal authority”, (iii) “relocation and reconstruction”, (iv) “living with family, friends or relatives”, and (v) “other”.

### Mental health

Finally, mental health was assessed using the Kessler-6 (K6) scale,^12^^,^^13^ which consists of six items (eg, “the following questions ask about how you have been feeling…during the past one month”: “(i) nervous… (ii) hopeless…(iii)…restless or fidgety…(iv) so depressed that nothing could cheer you up…(v)…that everything was an effort…[or] (vi) worthless”). Each item was measured on a five-point scale from 0 to 4 (0 = “none of the time,” 1 = “a little of the time,” 2 = “some of the time,” 3 = “most of the time,” and 4 = “all of the time,”), and the total score ranged from 0 to 24. We classified respondents with score ≥5 as having some mental health problems and those with score 0–4 as not having any mental health problems.

### Marital status

Marital status was assessed by asking the question: “Do you have a partner?”. Participants were requested to select an answer from “unmarried”, “married”, “divorced”, and “widowed”.

### Statistical analysis

Our sample for analyses comprised 6,724 individuals (2,502 men and 4,222 women), after excluding participants who had missing responses in health checkups and the questionnaire. Poisson regression was used to evaluate the associations between poor dietary intake and the following independent variables: age (≥65 years [reference], <65 years), living conditions (acceptable [reference], rather difficult, difficult/severe), housing (the same as before the earthquake [reference], temporary housing, relocation and reconstruction, family, friends and relatives home, other), SC (group with high SC [reference], group with low SC), K6 (0–4 [reference], ≥5) and areas (Yamada [reference], Otsuchi, Kamaishi, Rikuzentakata) as independent variables. All analyses were stratified by gender because we determined a priori that the distribution of dietary intakes differed between men and women in our data. However, the interaction term between gender and SC on dietary intake was not statistically significant (*P* = 0.16). We estimated adjusted prevalence ratios (PR) and 95% confidence intervals (CIs). All analyses were conducted using SPSS version 24 (IBM Corp., Armonk, NY, USA), and *P* values less than 0.05 were regarded as statistically significant.

### Ethical considerations

This study was approved by the institutional ethics multiple committees of Iwate Medical University.

## RESULTS

Table 2 shows the participants’ characteristics. A total of 57.4% of the study population were aged 65 years or older, 31.6% were classified as having poor dietary intake, 17.2% answered that their living conditions were difficult/severe, 30.5% were living in temporary housing, 18.9% were in the low SC group, and 28.5% had some mental health problems.

**Table 2.** Characteristics of participants (*n* = 6,724)

		*n*	%
Gender	Men	2,502	37.2
	Women	4,222	62.8
Age group, years	≥65	3,860	57.4
	<65	2,864	42.6
Dietary intakes	Good dietary intake	4,598	68.4
	Poor dietary intake	2,126	31.6
Living conditions	Acceptable	3,953	58.8
	Rather difficult	1,615	24.0
	Difficult/severe	1,156	17.2
Housing	The same as before the earthquake	3,939	58.6
	Temporary housing	2,053	30.5
	Relocation and reconstruction	544	8.1
	Family, friends and relatives home	77	1.1
	Other	111	1.7
Social Capital (SC)	Group with high SC (4–10)	5,453	81.1
	Group with low SC (11–20)	1,271	18.9
K6	Good mental health (0–4)	4,811	71.5
	Poor mental health (≥5)	1,913	28.5
Marital status	Married	4,894	72.8
	Divorced	231	3.4
	Widowed	1,073	16.0
	Unmarried	526	7.8

K6, Kessler-6.

Participants who fulfilled the following criteria were considered as having good dietary intake: staple food item intake ≥three times a day; meat, fish and shellfish, eggs, or soybean products ≥twice a day; vegetables ≥twice a day; and fruit or dairy products ≥once a day.

All remaining participants were categorized as having poor dietary intake.

Dietary intakes according to age group and gender are shown in Table 3. We confirmed the significant differences in dietary intakes according to age in both men and women. Percentages of good dietary intake were higher among the older participants.

**Table 3.** Dietary intakes by age group and gender

	Age, years	Total	Good dietary intake		Poor dietary intake	
	
			*n*	%	*n*	%
Men	≥65	1,628	1,105	67.9	523	32.1
	<65	874	403	46.1	471	53.9
	Total	2,502	1,508	60.3	994	39.7

Women	≥65	2,232	1,782	79.8	450	20.2
	<65	1,990	1,308	65.7	682	34.3
	Total	4,222	3,090	73.2	1,132	26.8

Participants who fulfilled the following criteria were considered as having good dietary intake: staple food item intake ≥three times a day; meat, fish and shellfish, eggs, or soybean products ≥twice a day; vegetables ≥twice a day; and fruit or dairy products ≥once a day.

All remaining participants were categorized as having poor dietary intake.

Poisson regression analyses showed that younger respondents (aged <65 years) were more likely to report poor dietary intake (PR 1.48; 95% CI, 1.29–1.71 among men and PR 1.55; 95% CI, 1.36–1.77 among women) compared with older respondents (Table 4). Respondents were also significantly more likely to report poor dietary intake if they reported difficulties in living conditions (PR 1.18; 95% CI, 1.00–1.39 among men and PR 1.19; 95% CI, 1.01–1.40 among women). In addition, women with low perceptions of community SC were more likely to report poor dietary intake (PR 1.20; 95% CI, 1.04–1.38); however, the same associations were not observed among men. In sensitivity analyses, we also categorized SC into tertiles and quartiles and confirmed that there was no significant difference between SC and poor dietary intake among men, while we continued to observe a dose-response relationship among women. We found no significant difference in poor dietary intake and housing and mental health in either gender. Residents of Rikuzentakata were also less likely to report poor dietary intake (PR 0.58; 95% CI, 0.50–0.68 among men and PR 0.51; 95% CI, 0.44–0.59 among women).

**Table 4.** Prevalence ratios for poor dietary intake and related factors using Poisson’s regression analyses

	Men (*n* = 2,502)		Women (*n* = 4,222)	
	
	PR	95% CI	PR	95% CI
Age group, years				
≥65	1.00	(reference)	1.00	(reference)
<65	1.48	(1.29–1.71)	1.55	(1.36–1.77)
Living conditions				
Acceptable	1.00	(reference)	1.00	(reference)
Rather difficult	1.09	(0.93–1.27)	1.12	(0.97–1.30)
Difficult/severe	1.18	(1.00–1.39)	1.19	(1.01–1.40)
Housing				
The same as before the earthquake	1.00	(reference)	1.00	(reference)
Temporary housing	1.06	(0.92–1.22)	1.11	(0.97–1.30)
Relocation and reconstruction	1.03	(0.81–1.32)	0.99	(0.78–1.25)
Family, friends and relatives home	0.80	(0.43–1.50)	0.95	(0.56–1.62)
Other	0.82	(0.51–1.31)	1.07	(0.67–1.72)
SC				
Group with high SC (4–10)	1.00	(reference)	1.00	(reference)
Group with low SC (11–20)	1.06	(0.91–1.23)	1.20	(1.04–1.38)
K6				
Good mental health (0–4)	1.00	(reference)	1.00	(reference)
Poor mental health (≥5)	1.14	(0.98–1.32)	1.09	(0.96–1.23)
Marital status				
Married	1.00	(reference)		(reference)
Divorced	1.42	(1.05–1.93)	1.34	(1.03–1.75)
Widowed	1.49	(1.20–1.84)	1.21	(1.03–1.42)
Unmarried	1.20	(1.00–1.45)	1.49	(1.22–1.82)
Areas				
Yamada	1.00	(reference)	1.00	(reference)
Otsuchi	1.04	(0.89–1.22)	0.99	(0.86–1.15)
Kamaishi	1.34	(0.92–1.95)	0.99	(0.69–1.42)
Rikuzentakata	0.58	(0.50–0.68)	0.51	(0.44–0.59)

CI, confidence interval; K6, Kessler-6; PR, prevalence ratio; SC, social capital.

Participants who fulfilled the following criteria were considered as having good dietary intake: staple food item intake ≥three times a day; meat, fish and shellfish, eggs, or soybean products ≥twice a day; vegetables ≥twice a day; and fruit or dairy products ≥once a day.

All remaining participants were categorized as having poor dietary intake.

PR were adjusted for marital status and residential areas.

## DISCUSSION

This study revealed that poor dietary intake remains prevalent among survivors up to 3 years after the Great East Japan Earthquake and Tsunami. Risk factors for poor dietary intake included having an age <65 years, experiencing economic difficulties, and having low perceptions of community SC. To our knowledge, this is the first study to report that a low level of community SC is associated with poor dietary intake among survivors of natural disaster. Among the four items making up the SC index, the item “most people in this neighborhood are likely to help each other” was most strongly correlated with dietary intakes.

In previous reports, investigators have found correlations between SC and dietary intakes in the general population.^14^^,^^15^ For example, a study in a rural Japanese community in the northern part of Akita prefecture found that low SC correlated with low vegetable intake. The hypothesized reason for this association was attributed to people sharing their meals or eating together with their families or neighbors in rural areas.^15^ Since the disaster-affected areas in our study were also rural, we hypothesized that the same mechanism could apply.

In our study, SC was not correlated with poor dietary intake in men, suggesting that there may be gender differences in the relationship between SC and dietary intakes. Although men were much more likely to report poor dietary intake compared with women, the correlation between low SC and dietary intake was statistically significant only among women. Previous studies reported that women are more likely to engage in social community.^16^^,^^17^ In Japanese society, it is still quite common for women to be homemakers, so many women are responsible for the household’s grocery shopping and food preparation. Women’s social lives may have been more severely disrupted by the disaster because the opportunities to eat and to shop for groceries together with neighbors diminished considerably. However, the interaction between gender and SC on dietary intake was not statistically significant.

Previous research indicated that the destruction of homes in the earthquake/tsunami and subsequent residential relocation was associated with a drop in SC among survivors.^18^ Hence, we checked the interaction of housing damage and SC on dietary intakes. However, the interaction term was not statistically significant. We interpret this to mean that change in SC was due to other factors, such as losing close friends, rather than damage to people’s homes.

The present study found there was no statistically significant difference between housing and dietary intakes. It was inferred that dietary intakes change depending not on the type of housing, but rather the distance from housing to food access.

Another study found associations of cohabitation status with dietary behaviors as well as obesity, and the authors of that study suggested that men who ate alone and lived alone were more likely to skip meals and be obese.^19^ It has been shown that changing one’s dietary behavior depends on interactions with other people, suggesting that poor dietary intake could result from living alone or skipping meals. Younger adults were also reported to be more likely to skip meals.^20^ The percentage of people skipping breakfast was highest at 20 years of age in both men and women (30.0% of men and 25.4% of women).^21^ This suggests that living alone or skipping meals may result in poor dietary intake.

We also found notable variations in dietary patterns among our four field sites. Among our field locations, residents of Rikuzentakata demonstrated relatively good dietary intake. Compared with other regions, residents of Rikuzentakata had a higher intake of vegetables, possibly because agricultural activities resumed shortly after the earthquake.^22^ In addition, the prevalence of cerebrovascular disease in Rikuzentakata has been reported to be lower than that in the rest of Iwate Prefecture,^23^ except during the first 4 weeks after the Great East Japan Earthquake and Tsunami.^24^

Our study has several limitations. First, we did not examine the intake of specific nutrients; instead, our focus was on an overall index of “good dietary intake”. In our study, dietary intakes were evaluated using an index of food consumption frequencies based on the Japanese Food Guide.^9^ Previously, a similar index of good dietary intake was shown to predict mortality.^11^ Second, we were unable to investigate changes in the local food environment. The widespread destruction of property (including food retail stores) and the displacement of nearly a quarter of a million people (due to property damage) resulted in marked changes in the local food environment, which likely affected people’s food choices. A recent study reported an association between distance to retail stores and the risk of becoming house-bound among older survivors of Rikuzentakata.^25^ This underscores the importance of understanding the impact of changes in the local built environment, in addition to studying changes in the social environment after a disaster. Having retail stores and bus stops in closer proximity to residential areas may help not only to prevent social withdrawal among the older people, but also promote better dietary intakes. Third, we are unable to comment on whether the experience of hardship after a disaster is associated with specific dietary patterns, such as excess intake of unhealthy foods (eg, packaged processed foods, such as instant noodles) or poor intake of healthy foods (such as fresh vegetables). Fourth, we are not able to control for other possible confounders, such as education and health conditions (eg, diseases). Finally, we cannot infer causality owing to the cross-sectional nature of our data.

### Conclusion

We found that the prevalence of poor dietary intake was higher among survivors of the 2011 earthquake and tsunami than the general situation. In turn, we considered that perception of community SC was needed to maintain healthy intakes in women. It is important to keep SC and to prepare an environment that is easy to rebuild SC in the rural disaster areas to encourage healthy lifestyles.
